# Acute lymphoblastic leukemia diagnosis using machine learning techniques based on selected features

**DOI:** 10.1038/s41598-025-12361-4

**Published:** 2025-08-01

**Authors:** Enas M.F. El Houby

**Affiliations:** Systems & Information Department, National Research Centre, Dokki, Cairo, 12311 Egypt

**Keywords:** Acute lymphoblastic leukemia, Computer-aided diagnosis, Machine learning techniques, Cancer, Computational biology and bioinformatics, Engineering

## Abstract

Cancer is considered one of the deadliest diseases worldwide. Early detection of cancer can significantly improve patient survival rates. In recent years, computer-aided diagnosis (CAD) systems have been increasingly employed in cancer diagnosis through various medical image modalities. These systems play a critical role in enhancing diagnostic accuracy, reducing physician workload, providing consistent second opinions, and contributing to the efficiency of the medical industry. Acute lymphoblastic leukemia (ALL) is a fast-progressing blood cancer that primarily affects children but can also occur in adults. Early and accurate diagnosis of ALL is crucial for effective treatment and improved outcomes, making it a vital area for CAD system development. In this research, a CAD system for ALL diagnosis has been developed. It contains four phases which are preprocessing, segmentation, feature extraction and selection phase, and classification of suspicious regions as normal or abnormal. The proposed system was applied to microscopic blood images to classify each case as ALL or normal. Three classifiers which are Naïve Bayes (NB), Support Vector Machine (SVM) and K-nearest Neighbor (K-NN) were utilized to classify the images based on selected features. Ant Colony Optimization (ACO) was combined with the classifiers as a feature selection method to identify the optimal subset of features among the extracted features from segmented cell parts that yield the highest classification accuracy. The NB classifier achieved the best performance, with accuracy, sensitivity, and specificity of 96.15%, 97.56, and 94.59%, respectively.

## Introduction

Cancer remains a leading cause of death worldwide, responsible for about 10 million deaths in 2020, or approximately one in six deaths^1^. One of the most common types of cancer in children is blood cancer, or leukemia, which can be described as the excessive production of white blood cells (WBCs) in bone marrow by the immune system. The most common type of leukemia in children is acute lymphoblastic leukemia (ALL). It always infects young children and adults, particularly those over 50 years^2,3^. Early detection of cancer is important for a rapid response and enhancement of the rate of cure, but it is difficult because the symptoms of the cancer are absent at the beginning. Therefore, cancer remains one of the health topics, where several researchers try to contribute to improve the diagnosis, prevention and treatment. The time of diagnosis and finding effective treatment are considered big challenges^4–6^.

Traditionally, the diagnosis of Leukemia has relied on visual examination of microscopic blood and bone marrow samples. Manual examination may lead to inconsistent and non-standardized reports, which may contribute to potential misdiagnosis or delayed treatment. Therefore, an automated diagnostic method is essential to improve accuracy and reduce variability that may result from differences in physicians’ and hematologists’ experience levels, as well as operator fatigue. CAD systems based on machine learning techniques (MLTs) can provide promising solutions by automating the analysis of blood sample images and diagnosing them as normal or abnormal^4–6^.

The advancements in technology and artificial intelligence (AI) have paved the way for innovative solutions in medical diagnostics. AI encompasses a broad range of techniques designed to simulate human-like cognitive functions, such as learning and pattern recognition, to support complex data analysis and decision-making in fields like medical diagnostics. Data mining, a core subfield of AI, is specifically concerned with discovering patterns and correlations within large datasets and often relies on MLTs to extract useful knowledge from data. MLTs perform best when trained on substantial amounts of data. Large datasets help ensure that the models learn representative patterns and generalize well, which is critical in complex domains as medical diagnostics. In the context of CAD systems, data mining plays a critical role by utilizing these techniques to analyze medical images and data, ultimately extracting valuable patterns that enhance diagnostic accuracy^7,8^.

Therefore, CAD systems can serve as effective tools for the early detection of ALL using microscopic blood smear images. A typical CAD system consists of four main phases: preprocessing, segmentation of regions of interest (ROIs), feature extraction and selection, and classification. Each phase plays a vital role in accurately identifying leukemic cells, with preprocessing enhances image quality, while the subsequent phases enable detailed analysis and classification. By automating the diagnostic process, CAD systems enhance physicians’ performance by highlighting abnormal regions and providing quantitative analysis. This results in objective, consistent, and efficient support for physicians and pathologists, while reducing dependence on manual examination^7–9^.

Preprocessing is a crucial initial phase in CAD systems, as it prepares medical images for the subsequent analysis phases. During preprocessing, raw data are enhanced to improve the quality of the image and reduce noise and other distortions that could interfere with later phases of analysis. Techniques like image noise reduction, normalization, adjusting image intensity, and calculating the complement of the image are commonly applied to ensure that the images are clear and consistent, providing a more accurate foundation for segmentation and feature extraction. This phase is essential as poor-quality input data can lead to inaccurate feature selection and classification, undermining the overall performance of the CAD system^7,8^.

Segmentation is a vital phase in CAD systems, aiming to divide medical images into meaningful regions like organs or tumors. This phase enhances diagnostic accuracy by allowing focused analysis, aiding in tasks such as feature extraction, classification, and measurement. Effective segmentation is essential, as poor results can lead to misdiagnoses, making it a key factor in the reliability and success of CAD systems in clinical settings^5^.

Feature selection is an essential process for data mining and pattern recognition, especially when working with high-dimensional datasets, such as those found in medical imaging and diagnostics. While feature selection is a fundamental process in many machine learning applications, in the context of ALL diagnosis using CAD systems, its significance is heightened due to the complexity and high dimensionality of medical images and associated data. In medical diagnostics, the volume of features (such as shape features, texture features, etc.) can be large, and selecting the most relevant features for accurate classification is crucial not only to reduces the dataset’s complexity and so reduce computational complexity but also enhances diagnostic performance by eliminating irrelevant or redundant features that negatively impact the results of classification algorithms. Therefore, effective feature selection directly contributes to improving both the speed and accuracy of the diagnosis process, making it a key component of the system’s success^9,10^. Feature selection is a combinatorial optimization problem. Heuristic search algorithms are commonly employed to address this challenge, as they can select the optimal feature set more efficiently than exhaustive search methods, which require significant time. Of these are the evolutionary computation algorithms such as genetic algorithm, Ant Colony Optimization (ACO), firefly, and particle swarm optimization^11^.

The classification phase in CAD systems is crucial for converting segmented image data into diagnostic insights. During classification, the system analyzes the features extracted from the segmented regions and assigns them to predefined categories (e.g., benign vs. malignant). Accurate classification is vital for reliable diagnoses and decision-making. Using machine learning and pattern recognition, this phase ensures consistent, objective predictions, aiding clinicians, reducing errors, and enhancing diagnostic efficiency and patient outcomes^5^.

In this research, a CAD system for ALL has been proposed to classify each case in microscopic blood images as normal or abnormal. The ACO approach has been used for feature selection in conjunction with 3 different classifiers. ACO presents a feature selection tool inspired by the ants’ behavior and their ability to find the shortest path between a food source and the nest. The objective of this research is to find the minimum set of features that provides the best classification accuracy for ALL datasets.

This research contributes to the field of cancer diagnosis by developing an integrated CAD system that addresses common limitations in existing approaches, such as suboptimal feature selection and low performance of classifiers. By employing ACO for feature selection and comparing multiple classifiers, the system aims to achieve high diagnostic accuracy and robustness. The proposed CAD system has the potential to be integrated into clinical workflows as a decision-support tool, assisting hematologists and laboratory technicians in the early and reliable diagnosis of ALL from blood smear images. Such a tool can integrate AI-based tools into routine diagnostic workflows and thus reduce diagnostic time, improve accuracy in resource-limited settings, and ultimately enhance patient outcomes.

The remainder of this paper is organized as follows. Sect. “Related work” provides an overview of previous works related to CAD systems for diagnosis of Leukemia using medical images. Sect. “Materials and method” describes the used dataset and the proposed CAD system for ALL. Sect. “Experimental results and analysis” presents the experimental setup and results, while Sect. “Conclusion and future work” concludes the paper and discusses future directions.

## Related work

Computer-aided diagnosis has become an active research area; therefore, recently many researchers investigated different techniques for disease diagnosis. Among these diseases, cancer, particularly Leukemia, has attracted substantial attention from the research community due to its severity. The following subsections highlight some of the recent work focused on using CAD systems to diagnose Leukemia.

Alsaykhan & Maashi^5^ developed a hybrid model combining support vector machine (SVM) with particle swarm optimization to detect ALL. The SVM alone achieved an accuracy of 74.43%, while the hybrid SVM-PSO model achieved an accuracy of 93.14% using ALL-IDB benchmark dataset of microscope images.

Abhishek, et al.^12^ proposed a multi-level classification approach for acute leukemia using transfer learning. By extracting features using ResNet50, the SVM achieved an accuracy of 95%, while logistic regression achieved an accuracy of 96%. When MobileNet was used to extract features, an accuracy of 95% was achieved by k-NN classifier using K = 5.

Wang, et al.^13^ proposed a deep learning model based on the pre-trained Resnet 50 architecture to detect Acute Promyelocytic Leukemia in bone marrow smears. The model performed binary classification to differentiate between myelodysplastic syndromes (MDS) and non-MDS cases, with an accuracy of 91.4%. It was also used for three-class classification, identifying aplastic anemia, myelodysplastic syndromes, and acute myeloid leukemia with an accuracy of 92.9%.

Anilkumar et al.^14^ used two pre-trained deep learning Convolutional Neural Networks (CNNs), LeukNet and AlexNet, to classify ALL into B-cell and T-cell images without the need for image segmentation or feature extraction. The CNNs were applied to images from American Society of Haematology bank, achieving an accuracy of 94.12% using LeukNet and 91.18% using AlexNet.

Tuba, E., et al.,^15^ proposed an automatic method to detect ALL by classifying white blood cells as blasts or normal. Texture and shape features were represented as vectors and introduced to SVM as input. Bare bones fireworks algorithm was used to optimize SVM. It achieved an accuracy of 91.84%, sensitivity of 94.21% and specificity of 89.37% using the ALL-IDB dataset images.

Zhou, et al.^16^ introduced a CNN deep learning model to classify bone marrow raw images without pre-processing. Applied to a dataset of 1,732 images, the model achieved an accuracy of 89%, sensitivity of 86%, and specificity of 95%.

Kishore Babu, et al.^6^ developed a CAD system for Leukemia detection using images from the publicly available Kaggle dataset. The system could distinguish among white blood cells, red blood cells, and platelets. Features relevant to lymphocyte cell shape and color were extracted, and the selected features were used to classify cells using SVM classifier into normal and blast.

Chen, et al.^17^ developed a Resnet101-9 ensemble model using an approach to find the combinations of fine-tuning model hyperparameters for the pre-trained Resnet-101 transfer learning model that achieve the best accuracy in classifying ALL in microscopy images. The model achieved an accuracy of 85.11% and an F1-score of 88.94 using C-NMC^18^ dataset.

Billah& Javed^19^ proposed a Bayesian Convolution Neural Network (BCNN)-based for classifying ALL microscopic images of blood samples. Multiple architectures were explored, with the best achieving an accuracy of 94%, sensitivity of 89.33, and specificity of 99.33 using the ALL-IDB public image dataset.

Pervez, et al.^20^, proposed a framework that utilizes EfficientNetB3 for ALL classification, incorporating explainability techniques. The approach is based on federated learning, which trains models across different organizations while keeping patient information decentralized and encrypted. It achieved an accuracy of 96.5%, an F1-score of 94.4%, a sensitivity of 0.923, and an AUC of 0.98 using a publicly available dataset that contains 15,135 images.

While previous studies have demonstrated promising results in the diagnosis of Leukemia using various MLTs and deep learning techniques, several limitations remain. For instance, some approaches overlook essential preprocessing steps, which can negatively affect model performance. Additionally, feature selection is often underexplored, leading to the inclusion of redundant or irrelevant features that may degrade classification accuracy. These gaps highlight the need for a more integrated approach that encompasses proper preprocessing, segmentation, effective feature extraction and selection, and robust classification for ALL diagnosis.

To address these limitations, this research proposes a comprehensive system that integrates all four phases, with particular emphasis on feature selection. Specifically, an ACO algorithm has been employed to identify the optimal subset of features extracted from segmented cell components, aiming to maximize classification accuracy while reducing feature dimensionality. Furthermore, multiple classifiers have been evaluated to compare their performance, and the classifier that achieves the best performance with the selected features is chosen as the final model.

## Materials and methods

In this research, a computer aided diagnosis (CAD) system for Acute Lymphoblastic Leukemia (ALL) based on microscopic images has been developed. The next subsections illustrate the used dataset and the structure of the proposed CAD system, which includes different phases to classify microscopic blood smear images.

### Materials

The ALL-IDB^21^ dataset for ALL was employed for this study. The ALL-IDB dataset is a publicly available blood smear images dataset. Each image is in JPG format with resolution of 2592 × 1944 pixels at 24 bit of color depth. The dataset includes samples from both ALL patients and healthy individuals, making it an appropriate representation of the variability that a CAD system needs to handle.

The ALL-IDB database includes 2 various subsets which are ALL-IDB1 and ALL-IDB2. ALL-IDB1 contains 108 images of both ALL patients’ images and healthy ones. While ALL-IDB2 includes 260 images, each image is one of the cropped areas of interest containing either normal or blast cells which are cropped from ALL-IDB1dataset. The ALL-IDB dataset has been widely used in the literature for the development of machine learning-based diagnostic tools, ensuring that the results of the current study are comparable and benchmarked against previous work in the field.

### The proposed CAD system

The proposed CAD system includes 4 phases which are preprocessing, segmentation of the region of interest (ROI), features extraction and selection, and finally classification phase. Each phase plays a crucial role in the accurate diagnosis of ALL from the blood smear images. The following subsections will provide a detailed explanation of each phase.

#### Preprocessing phase

In the preprocessing phase, different steps are applied to the microscopic blood smear images to prepare them for the next phases, they are as follows:

- The images are converted to grayscale using the standard RGB-to-grayscale transformation to simplify the image and reduce computational complexity.
- The intensity is adjusted to enhance contrast.
- The images are inverted using a complement operation to enhance contrast between the foreground (cells) and background.

**Fig. 1:**
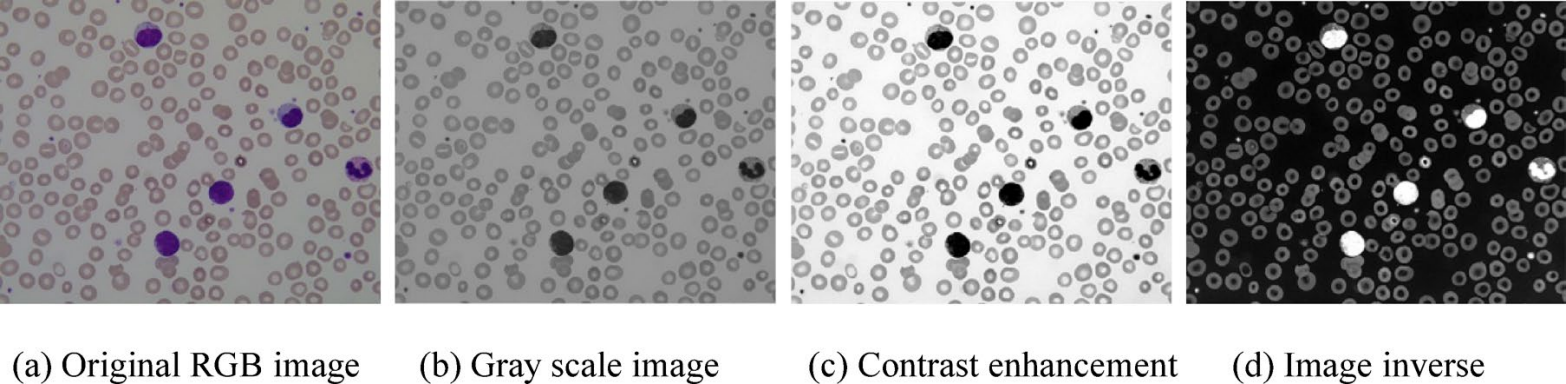
Visual results of preprocessing steps applied to a microscopic blood smear image.

These preprocessing steps are essential for improving image quality and ensuring accurate analysis. Converting the image to grayscale reduces computational complexity by removing color information while preserving structural details. Grayscale images contain only intensity information, which is often enough for medical image analysis. This simplification speeds up processing and makes subsequent steps like contrast enhancement more efficient and accurate. Contrast enhancement through intensity adjustments makes key features, such as cell boundaries and internal structures, more distinguishable from the background. This step improves the visibility of important details, which in turn helps with more accurate segmentation and analysis in later phases. Image inversion using complement operation is applied during preprocessing to enhance the contrast between cells (leukocytes) and the background, which aids in accurate segmentation and feature extraction. This step makes the cells more distinguishable, especially in low-contrast images, and ensures consistent highlighting of features despite variations in image intensity or staining. Ultimately, these preprocessing steps contribute to more reliable, classification and diagnosis. The results of applying these preprocessing steps to a microscopic blood smear image are shown in Fig. 1.

#### Segmentation phase

**Fig. 2:**
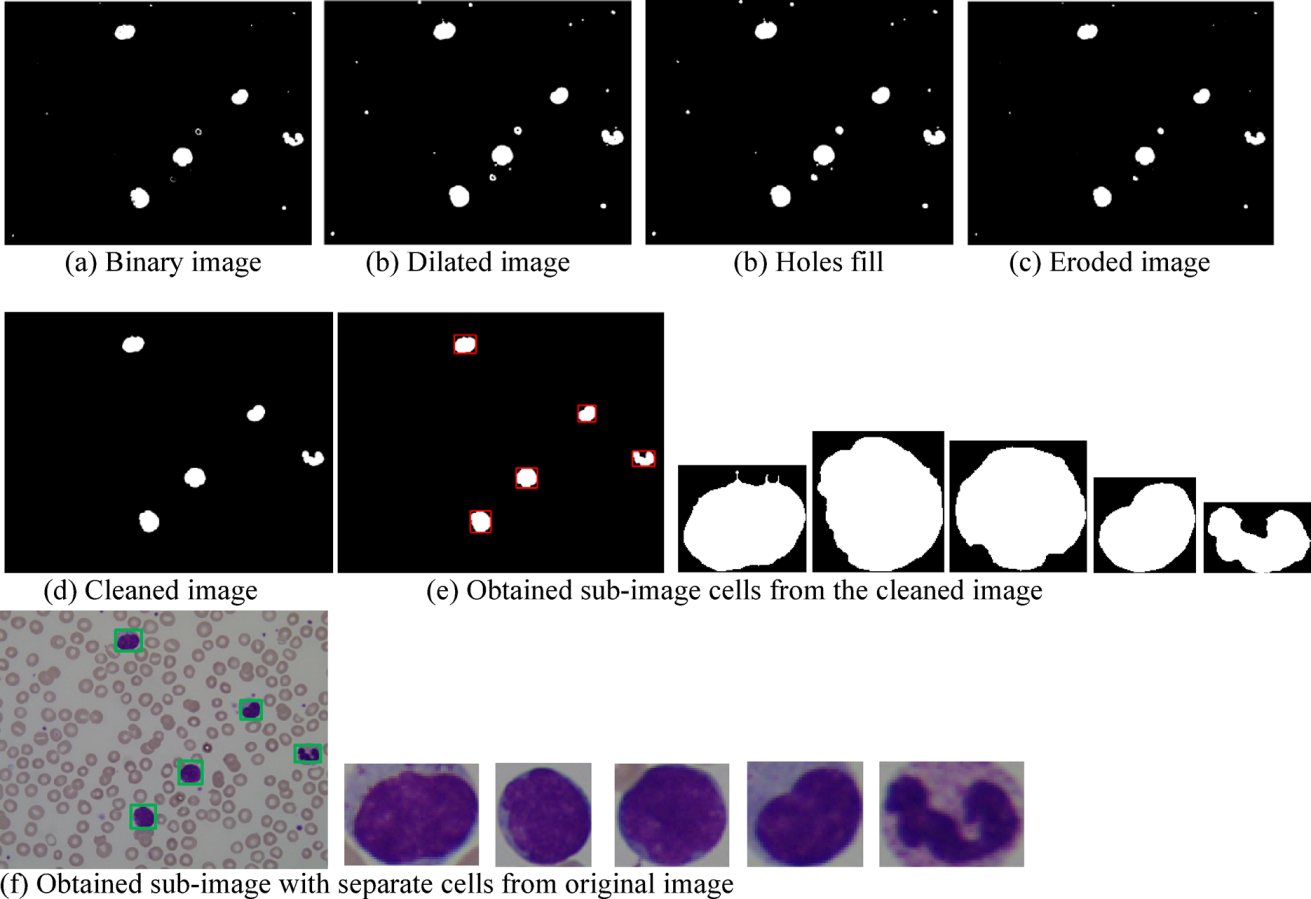
Blood cell segmentation

In the segmentation phase, suspected regions of interest (ROI) are cropped from the preprocessed image. These segmented ROI are then used for extracting different features that can differentiate between normal and abnormal regions. Figure 2 illustrates key steps of the segmentation process, including:

- The preprocessed image is first converted to binary format using adaptive thresholding, which distinguishes cells (leukocytes) from the background based on intensity.
- Morphological operations such as dilation, hole filling, and erosion are applied to refine cell shapes and separate overlapping regions.
- Connected components analysis is performed to identify distinct cell-like regions.
- Small components, likely corresponding to noise or irrelevant artifacts, are filtered out based on a predefined area threshold.
- Bounding boxes are drawn around each of the remaining connected components to localize individual cells.
- These bounding boxes are then used to crop ROIs from the original image, where each ROI contains a single cell.
- Further segmentation is performed within each ROI to isolate the nucleus and cytoplasm, based on intensity and color characteristics, as outlined in^22^.

The segmentation of ROI is performed using some crucial steps. Binary conversion is applied to the preprocessed image to convert the image to a binary format where the cells (leukocytes) and background are distinguished based on intensity levels. This binary image helps in isolating regions containing potential cells of interest. Then, the morphological operations like dilation, hole filling and erosion are applied to refine the binary image, closing small gaps in the cells and ensuring that the cells are correctly identified as separate regions. The next step is to identify individual connected components (i.e., clusters of pixels that correspond to cells or regions of interest). After calculating the connected components areas, those that are smaller than some specified area, are removed to clean the image. A bounding box is then drawn around each of the connected components in the cleaned image to isolate individual cells or regions that may be normal or abnormal. The segmented regions that correspond to each bounding box are cropped from the original image. Each cropped ROI contains one cell. Further segmentation steps are applied to isolate the nucleus, and cytoplasm from the cell, within each cropped ROI. These cropped images are extracted as sub-images and treated individually for further analysis, such as feature extraction and classification and diagnosis. It ensures that the CAD system accurately differentiates between normal and abnormal cells.

#### Features extraction and selection phase

In this phase, a variety of features, including shape, color, and texture, are extracted from the segmented cells, nuclei, and cytoplasm. The complete set of extracted features is shown in Table 1. In total, 66 features are extracted, which serve to characterize and differentiate between normal and abnormal cells. The most relevant features are selected for classification. The whole process can be summarized as follows:

- Various features are extracted from the segmented cells, nuclei, and cytoplasm. These features include:

- *Shape features* (e.g. area, perimeter, and elongation):

Extracted from the binary images of segmented regions using morphological analysis functions (e.g. regionprops) by analyzing the boundary and pixel distribution of the objects (cells, nuclei, or cytoplasm).

- *Texture features* (e.g. correlation and energy):

Extracted using the Gray-Level Co-occurrence Matrix (GLCM), which capture spatial relationships between pixel intensities. These features help characterize the internal texture patterns of the segmented regions.

- *Color features* (e.g. mean and standard deviation):

Computed from the RGB and HSV color channels of the original images. These features are extracted using statistical analysis of color histograms and distributions to reflect intensity and color variations in different regions.

- The most relevant features are selected using ACO to enhance classification accuracy.

The first step in this phase is to extract shape, texture, and color features from the segmented cells, nuclei, and cytoplasm. Shape, texture, and color features were chosen because they capture key morphological and visual characteristics that are commonly altered in abnormal or malignant cells. Shape features reflect structural irregularities often seen in abnormal nuclei and cytoplasm, such as nuclear enlargement or irregular contours. Texture features help quantify the internal patterns within the cell or nucleus, which can differ significantly between healthy and abnormal cells. Color features are useful in identifying staining intensity variations, which may correspond to cellular abnormalities. These feature types are widely used in the literature for cytological analysis because of their proven relevance in enhancing classification performance. By focusing on biologically and visually meaningful features, the model can effectively differentiate between normal and abnormal cells, improving diagnostic accuracy.

**Table 1:** The set of extracted features.

No.	Type	Features
1	Shape	Area, filled area, convex area, extent, major axis length, eccentricity, solidity, diameter, minor axis length, perimeter, orientation, Euler number, circularity, elongation
2	Texture	Correlation, energy, contrast, homogeneity
3	Color	Standard deviation, mean, entropy, variance

After feature extraction, the feature selection step has been used to select the most relevant features for classification. In this research, Ant Colony Optimization (ACO)^23^ hybridized with multiple classifiers has been used to select the most relevant features subsets for classification from all extracted features. The features subsets that achieve the highest performance have been selected from among various features subsets as recommended discriminate features subsets. ACO iteratively explores feature subsets by probabilistically moving from one feature to another, based on pheromone levels (τ) and heuristic values (η), guided by feedback from the classifiers. The ACO algorithm uses the following probabilistic transition function to select features (Eq. 1).

1 $$P_{i}^{k} \left( t \right) = \frac{{\left( {\tau _{i} \left( t \right)} \right)^{\alpha } \left( {\eta _{i} \left( t \right)} \right)^{\beta } }}{{\sum\nolimits_{{j \in N_{j}^{k} }} {\left( {\tau _{j} \left( t \right)} \right)^{\alpha } \left( {\eta _{j} \left( t \right)} \right)^{\beta } } }},j \in N_{j}^{k}$$

Where:

$\:{N}_{j}^{k}$ is the feasible neighborhood of the ant *k*, which are the features that have not been selected by ant *k.*

$\:{\eta\:}_{i\:\:}\left(t\right)$ is the heuristic information of the feature $\:\left(\text{i}\right)$ at the time $\:t$.

$\:{\tau\:}_{i}\left(t\right)$ is the pheromone value on the feature $\:\left(\text{i}\right)$ at the time $\:t$.

$\:\alpha\:$ and $\:\beta\:$ are weights which represent the relative effect of the pheromone $\:\tau\:\:$and the heuristic information $\:\eta\:$, respectively.

The pheromone levels $\:{\tau\:}_{i}\left(t\right)$ of different ants have been updated where pheromone quantities increase according to the pheromone updating equations which are as follows:

2 $$\:{\tau\:}_{i}\left(t+1\right)\:=\:{\tau\:}_{i}\left(t\right)\times\:\left(1\:-\rho\:\right)+\:\varDelta\:{\tau\:}_{i}\left(t\right)\:\:\:\:\:\:\:\:\:\:\:\:\:\:\:\:\:\:\:\:\:\:\:\:\:\:\:\:\:\:\:\:\:\:\:\:\:\:\:\:\:\:\:\:\:\:\:\:\:\:\:\:\:\:\:\:\:\:\:\:\:\:\:\:\:\:\:\:\:\:\:\:\:\:\:\:\:$$

3 $$\:\varDelta\:{\tau\:}_{i}^{k}\left(t\right)=\left(\begin{array}{cc}\:\:\:Q\:\:\:\:\:\:&\:\text{i}\text{f}\:\text{t}\text{h}\text{e}\:\text{f}\text{e}\text{a}\text{t}\text{u}\text{r}\text{e}\:\left(\text{i}\right)\:\text{i}\text{s}\:\text{c}\text{h}\text{o}\text{s}\text{e}\text{n}\:\text{b}\text{y}\:\text{t}\text{h}\text{e}\:\text{a}\text{n}\text{t}\:\text{k}\\\:0\:\:\:&\:\text{o}\text{t}\text{h}\text{e}\text{r}\text{w}\text{i}\text{s}\text{e}\end{array}\right)$$

where: *ρ* is the pheromone evaporation rate of the features (0 *< ρ <* 1), and.

$\:\varDelta\:{\tau\:}_{i}^{k}\:$is the pheromone deposited by the ant $\:k$ that found the best solution for the current iteration^24^.

The heuristic value has been calculated using the Relief approach criterion for feature selection^25^.

#### Classification phase

Different MLTs can be used as classifiers distinguish between normal and abnormal regions. In this research, Naive Bayes (NB), Support Vector Machine (SVM), and k-Nearest Neighbors (K-NN)^26^ are used to classify cases based on the selected subsets of features by ACO. For each classifier, the different subsets of generated features by ACO are iteratively introduced into the classifier, and the classifier performance is evaluated using each subset. Feedback from the classifier is then used to guide ACO in selecting the next optimal feature subsets. This iterative process continues until the highest possible classification accuracy is achieved for that classifier, identifying the most informative subsets of feature. The same process is repeated with the different classifiers. Ultimately, the classifier and corresponding feature subset that yield the best overall classification accuracy are selected. The whole process can be summarized as follows:

- ACO is used to generate different subsets of features.
- These feature subsets are iteratively introduced to a classifier.
- The classifier is evaluated based on its performance using each subset.
- ACO updates its selection strategy for the next optimal feature subsets based on performance feedback from the classifier.
- This process continues until the highest classification performance is achieved for the given classifier.
- The same iterative process is repeated for all classifiers.
- Finally, classification results are compared across all classifiers to determine the combination of classifier and feature subset that yields the best overall accuracy.

The use of multiple MLTs, such as NB, SVM, and K-NN, is based on the principle of model diversity, which can lead to improved classification performance. Different classifiers have unique strengths and weaknesses in terms of handling data distributions, decision boundaries, and computational complexity. By evaluating multiple classifiers, we aim to exploit their individual strengths and mitigate potential weaknesses, leading to a more robust and reliable CAD system. The performance of different classifiers can be evaluated in terms of accuracy, sensitivity, and specificity. The iterative process of using the ACO algorithm to select optimal feature subsets ensures that the performance of all classifiers is improved as much as possible. Ultimately, the classifier that yields the best performance with the selected features is chosen as the final classifier. In conclusion, the use of different MLTs allows for a comprehensive evaluation of various classification strategies, ensuring that the most effective model for diagnosing ALL from blood smear images is selected.

**Fig. 3:**
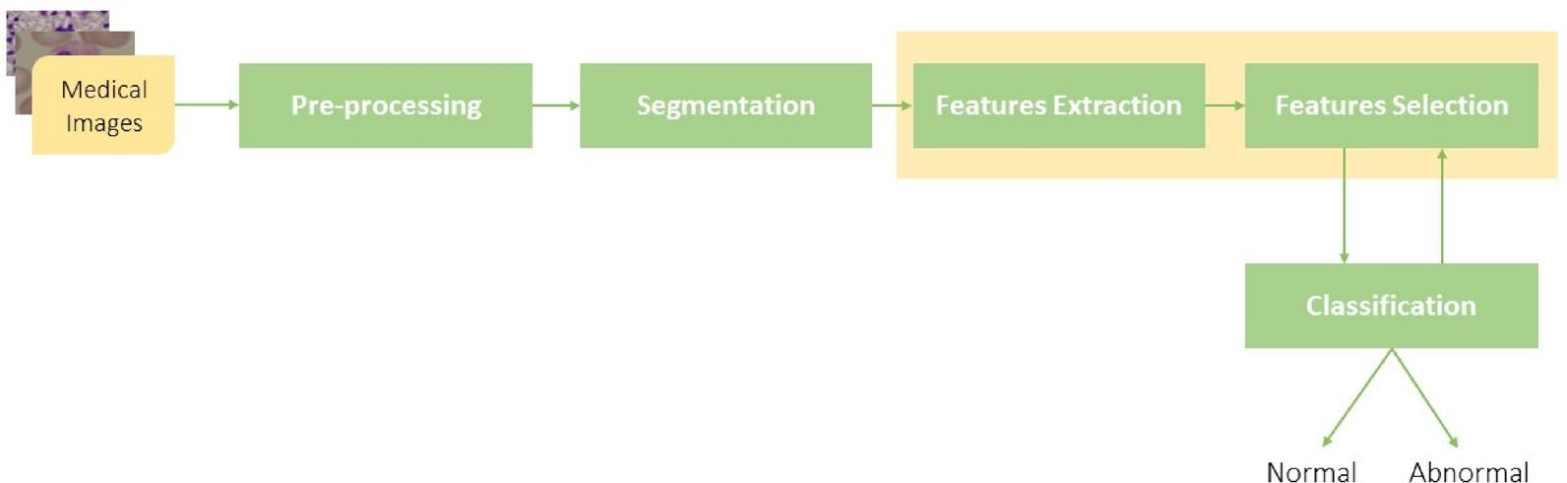
Block diagram of different phases of the proposed CAD system.

The block diagram of CAD system process has been summed up in Fig. 3; it illustrates different CAD phases and the sequences of these phases. In summary, the detection of disease (ALL) in the proposed system is achieved by combining these phases in a sequential manner: preprocessing for image preparation, segmentation for isolating ROI, feature extraction and selection for identifying important characteristics, and classification for detecting the disease. The final output is a classification result specifying whether the cell is normal or abnormal, indicating the potential presence of ALL.

## Experimental results and analysis

Extensive experiments have been conducted to get the best feature subsets selected by ACO with the various classifiers to achieve the highest possible accuracy. Matlab^®^ 2018 software on an Intel^®^Core™ i7-8550U CPU @ 1.8 GHz 1.99 GHz, 16.0 GB RAM computer has been used for implementation. The CAD system was tested using ALL-IDB2 dataset which has been labeled as normal or abnormal for ALL patients. The dataset was divided into 70% for training and 30% for testing. After applying the preprocessing phase and identifying ROI through segmentation, the segmented cells have been classified as normal or abnormal based on the selected features. Three different classifiers which are NB, SVM and K-NN were used. Each classifier works iteratively with ACO to select from the extracted features the best feature subset that achieve the highest possible accuracy.

**Fig. 4:**
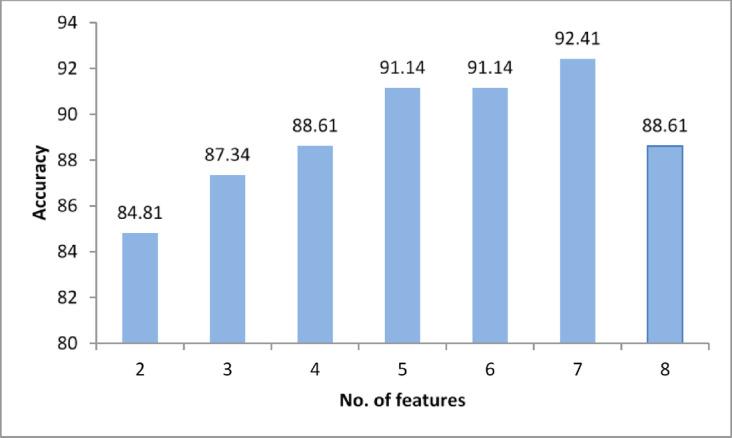
The achieved accuracy using K-NN versus number of features.

The number of extracted features is 22 features, and where these features are extracted from Cytoplasm, Nucleus, and cell, therefore the total number of extracted features is 66 features. The classifiers run for 100 generations using ACO, with each generation including 3 ants, yielding 300 different feature subsets for each number of selected features. The highest achieved accuracies using K-NN with the different numbers of selected feature subsets are presented in Table 2. As shown in Table 2, by increasing the number of selected features from 2 to 5 features, the accuracy increased. Increasing the number of selected features to 6 resulted in the same accuracy (91.14%) as with 5 features, while the accuracy peaked at 92.41% with 7 features. After that, it decreased to 88.61% with 8 features. Figure 4 shows the achieved accuracy using K-NN versus the number of selected feature subsets. According to the results, the highest accuracy has been achieved with a subset of 7 features, so it is the recommended subset to build K-NN classifier for this dataset. The selected features subset are “Energy” and “Entropy” from Cytoplasm, then the selected features from Nucleus are “Extent”, “Entropy”, and “Circularity”, and finally from Cell the selected features are “Circularity” and “Euler Number”.

**Table 2:** Sample Of selected features subsets that achieved the best accuracy/no. Of feature using K-NN.

No. of features	Accuracy %	Selected features subsets
2	84.81	Diameter_Nuclus_; mean_cell_
3	87.34	Solidity_Cytoplasm_; Elongation_Cytoplasm_; Circularity_Nuclus_
4	88.61	Area_Nuclus_; FilledArea_Cytoplasm_; ConvexArea_cell_; FilledArea_cell_
5	91.14	Entropy_Nuclus_; Entropy_Cytoplasm_; Circularity_cell_; EulerNumber_cell_; Circularity_Nuclus_
6	91.14	Entropy_Nuclus_; Extent_Nuclus_; Entropy_Cytoplasm_; Circularity_cell_; EulerNumber_cell_; Entropy_cell_
7	92.41	Energy_Cytoplasm_;Extent_Nuclus_;Entropy_Cytoplasm_;Circularity_cell_;Entropy_Nuclus_;Circularity_Nuclus_; EulerNumber_cell_
8	88.61	Eccentricity_Cytoplasm_; MinorAxisLength_cell_; mean_Nuclus_; Diameter_Nuclus_; MajorAxisLength_Nuclus_; mean_cell ;_ Diameter _Cytoplasm_; EulerNumber _cell ;_

**Fig. 5:**
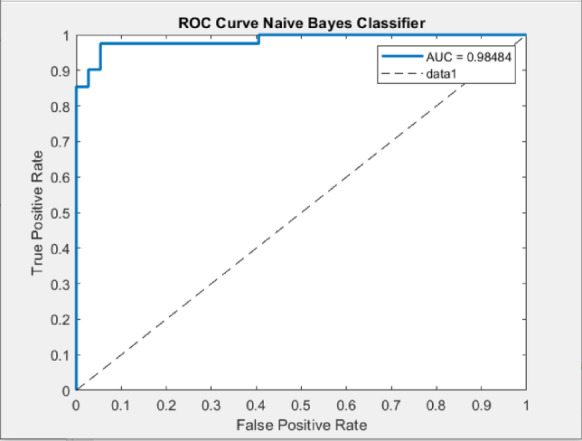
ROC curves for the NB classifier.

**Fig. 6:**
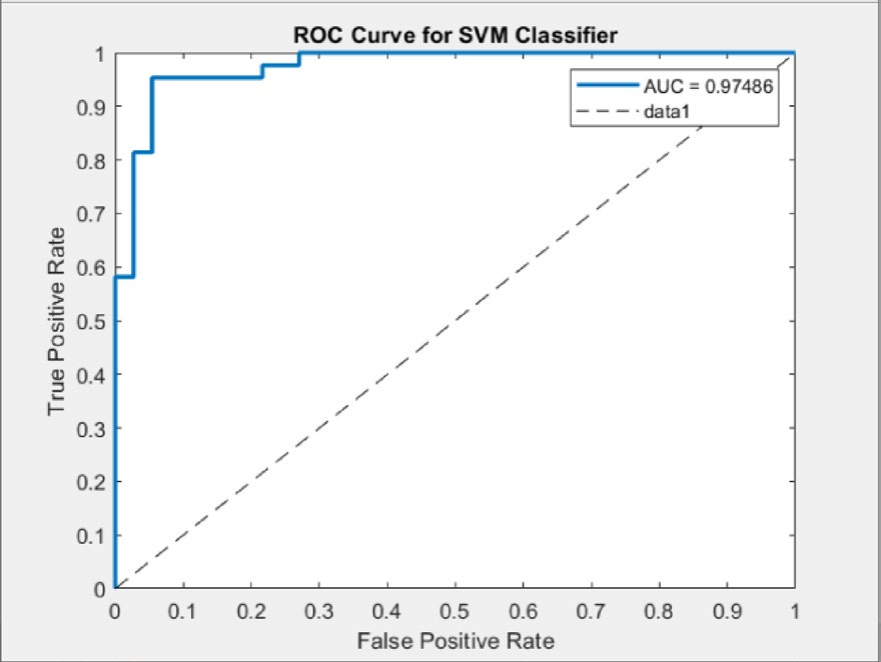
ROC curves for SVM classifier.

**Fig. 7:**
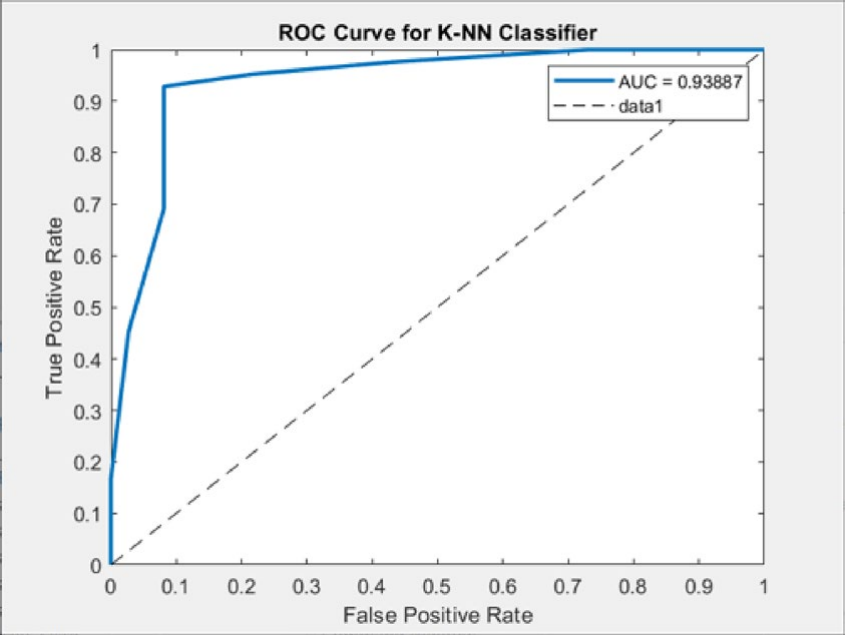
ROC curves for K-NN classifier.

**Fig. 8:**
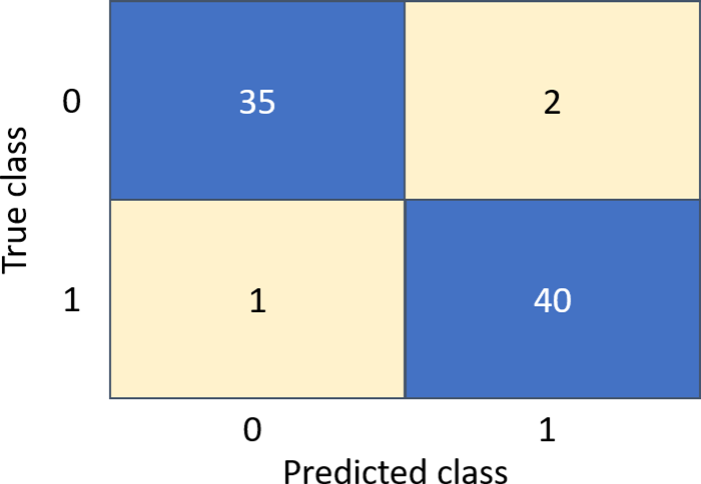
Confusion matrix of the best model (NB).

By applying the same process for the other classifiers, the best feature subsets that achieved the best performance have been captured. Table 3 illustrates the best classification accuracies achieved by the NB, SVM, and K-NN classifiers with the selected feature subsets that achieved these accuracies. Table 4 shows other metrics for the evaluation of the best achieved performance of the different classifiers using the selected features subset. Figures 5, 6 and 7 show the ROC curves for the best of each of the different classifiers. Figure 8 shows the confusion matrix of the classifier that yields the best performance overall which is NB.

**Table 3:** The selected features subset that achieved the best accuracy/no. Of feature for different classifiers.

Claasifier	No. of features	Accuracy	Selected features subsets
NB	6	96.15	Contrast_Nuclus_; Circularity_Cytoplasm_; Circularity_Nuclus_; Eccentricity_Cytoplasm_; Orientation_Nuclus_ ; Area_Cell_
SVM	5	93.75	EquivDiameter_Cytoplasm_; EquivDiameter_cell_; Std_Nuclus_; Eccentricity_cell_; MinorAxisLength_cell_
KNN	7	92.41	Energy_Cytoplasm_; Extent_Nuclus_; Entropy_Cytoplasm_; Circularity_cell_; Entropy_Nuclus_; Circularity_Nuclus_; EulerNumber_cell_

**Table 4:** The achieved performance using selected features subset.

Classifier	Accuracy %	Sensitivity %	Specificity %	Positive predictive value	Negative predictive value
NB	96.15	97.56	94.59	0.95	0.97
SVM	93.75	93.02	94.59	0.95	0.92
K-NN	92.41	92.86	91.89	0.93	0.92

The ROC analysis validates the classification performance of the models. The NB classifier achieved the highest AUC value of 0.9848, indicating excellent class separability. An AUC close to 1.0 illustrates that the model effectively distinguishes between the two classes and maintains a strong trade-off between sensitivity and specificity. The SVM classifier followed closely with an AUC of 0.97486, also reflecting strong separability, but slightly less than NB. In contrast, the KNN classifier achieved a comparatively lower AUC of 0.93887, which still indicates good performance.

Figure 8 shows that the number of true positives (TP) is 40, representing leukemia (ALL) cases correctly classified, while the number of true negative (TN) is 35 indicating normal cases correctly classified, False positive (FP) is 2 which the normal cases are incorrectly classified as ALL, and only 1 false negative (FN), meaning that only one ALL case is classified as normal. These results demonstrate the high diagnostic performance of the proposed CAD system, with very few misclassifications. The presence of only one FN out of 41 actual positive cases indicates high sensitivity, which is critical in medical diagnostics to ensure diseased cases are not missed. Similarly, the low number of FP reflects high specificity, minimizing unnecessary follow-up for healthy individuals.

To clarify the importance of feature selection and its impact on classification performance, the same classifiers have been used to classify ALL dataset without feature selection, and so all features have been used to classify the dataset. Table 5 compares the classification accuracy before and after feature selection using the various classifiers, showing the percentage of feature reduction of the selected feature subsets. Table 5 shows that all classifiers achieved better accuracy with selected features, even though the selected features are much less than the total features which are 66 features. For example, the NB classifier achieved 79.71% accuracy using all 66 features, but its accuracy increased to 96.15% when using the selected 6 features, representing only 9.1% of the total features. This results in a feature reduction of 90.9%.

**Table 5:** The percentage of features reduction and the achieved accuracy using different classifiers.

Classifier	Reduced subset	% Reduction	Accuracy of total features	Accuracy of selected features
NB	6	90.9%	79.71%	96.15%
SVM	5	92.42%	61.4%	93.75%
KNN	7	89.39%	84.21	92.41%

**Fig. 9:**
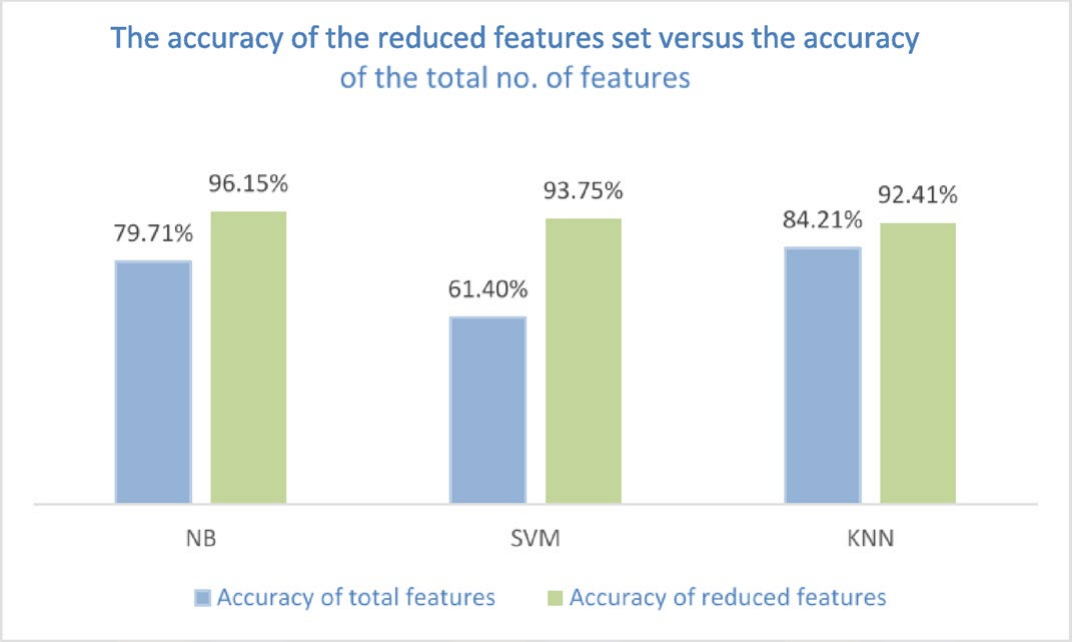
The comparison of the accuracy for the reduced feature set versus the accuracy of the total no. of features.

Figure 9 compares the accuracy achieved with all features versus the accuracy with selected features for the three classifiers. It is clear that the selected features achieve higher accuracy than the total number of features, which clarifies that the irrelevant and redundant features mislead the classifiers and therefore affect the classifier negatively.

As shown from the results, all classifiers achieved high classification performance, however, the NB achieved the best overall performance (AUC = 0.9848, accuracy = 96.15, sensitivity = 0.9756, specificity = 0.9459), demonstrating the effectiveness of the proposed CAD system in improving diagnostic performance. The integration of ACO for feature selection resulted in superior model performance compared to using the full feature set, confirming the importance of selecting relevant features for improving the classification performance and reducing the feature dimensionality. Furthermore, the comparative evaluation among NB, SVM (AUC = 0.9749), and KNN (AUC = 0.9389) emphasizes NB’s superior diagnostic capability. These findings validate the impact of the system’s design choices, such as optimized feature selection and classifier comparison. By improving efficiency and diagnostic reliability, the proposed CAD system supports the objective of assisting hematologists and lab technicians in the early detection of ALL.

**Table 6:** The comparison among the accuracy of the proposed method with previous works.

Reference	Classification technique	Acc. (%)	Sens. (%)	Spec.(%)
^5^	A hybrid model of SVM and PSO	93.14	-	-
^15^	A hybrid Bare bones fireworks and SVM.	91.84	94.21	89.37
^19^	Bayesian CNN (BCNN)-based	94	89.33	99.33
Proposed method	A hybrid ACO and K-NN	92.41	92.86	91.89
Proposed method	A hybrid ACO and SVM	93.75	93.02	**94.59**
Proposed method	A hybrid ACO and NB	**96.15**	**97.56**	**94.59**

The highest achieved values are given in bold.

Table 6 compares the performance achieved by the proposed work with other research that used the same dataset for fair comparison. It is clear that the proposed method outperforms the others although they used the same dataset and even when using the same classifier (SVM). Our proposed method achieved an accuracy of 93.75%, sensitivity of 93.02% and specificity of 94.59% using SVM. In contrast^5^, achieved an accuracy of 93.14% and^15^ achieved an accuracy of 91.84%, sensitivity of 94.21% and specificity of 89.37% using the same classifier (SVM). While^19^ achieved an accuracy of 94%, sensitivity of 89.33, and specificity of 99.33 using Bayesian Convolution Neural Network. Moreover, the performance of the proposed method was improved using NB classifier, achieving an accuracy of 96.15%, sensitivity of 97.56% and specificity of 94.59%.

The proposed method demonstrates improved performance compared to previous studies, primarily due to the use of ACO for selecting the most discriminative feature subsets. In this study, ACO outperforms PSO and Fireworks algorithms because of its strong global search capability and efficient exploration of the feature space through positive feedback and pheromone updating. Unlike PSO, which may converge prematurely, or Fireworks, which can be sensitive to parameter tuning, ACO effectively explores the search space to select optimal feature combinations and reinforces high-quality subsets over iterations. This leads to more stable, discriminative selections, enhancing the classifier’s ability to distinguish between normal and leukemic cells, thereby contributing to higher accuracy, sensitivity, and specificity observed in our CAD system.

## Conclusion and future work

In this research, a CAD system for acute lymphoblastic leukemia (ALL) diagnosis using microscopic blood smear images was developed. It contains four phases, which are preprocessing, segmentation of regions of interest, feature extraction and selection, and finally, classification. To enhance diagnostic accuracy and robustness, a hybrid approach combining Ant Colony Optimization (ACO) with multiple classifiers was employed for feature selection, which effectively reduced the 66 extracted features, covering cell, nucleus, and cytoplasm characteristics to the most relevant subset. This not only improved classification accuracy but also reduced computational complexity. Multiple classifiers were evaluated and compared, demonstrating that the Naïve Bayes model achieved the highest performance, with an accuracy of 96.15%, sensitivity of 97.56%, specificity of 94.59%, and AUC of 0.9848. These results confirm the effectiveness of the proposed system in accurately distinguishing between ALL and normal cases. They also validate the system’s design choices, such as the use of ACO for optimized feature selection and the comparative evaluation of classifiers, which align with the study’s objective of developing a robust, efficient, and accurate CAD system that can support hematologists and lab technicians in early and reliable ALL diagnosis.

In future work, we aim to apply the proposed CAD system to local Egyptian blood smear datasets to evaluate its generalizability and performance further. We will also explore the use of advanced segmentation techniques and incorporate more feature extraction and selection methods to further improve performance. Additionally, exploring deep learning and transfer learning-based models may further boost diagnostic accuracy. Lastly, we will investigate the potential of adapting the CAD system for diagnosing other types of cancers and diseases, broadening its applicability in the medical field.

## Data Availability

The data used has been obtained from an available online database and has been referenced in the manuscript.
